# Supplementary material for: The genome sequence of the Elm Groundling moth, Carpatolechia fugitivella (Zeller, 1839)
Source: Wellcome Open Res. 2024 Apr 23;9:211. [Version 1] doi: 10.12688/wellcomeopenres.21267.1 (PMC11320048; doi:10.12688/wellcomeopenres.21267.1)
Supplement: Peer review history

# Reviewer report: Reviewer response for version 1

**DOI:** 10.21956/wellcomeopenres.23522.r92046

**Published:** 2024-08-12

**Authors:** Jerome H L Hui

**Affiliations:** 1 The Chinese University of Hong Kong, Hong Kong, Hong Kong

In this data note, Boyes and colleagues reported the genomic resource of a male moth *Carpatolechia fugitivella *(Zeller, 1839) *.* According to the NatureSpot, UKMoths, and NBN, this species can be found in many places in Britain. Prior to this report, molecular data available for this species is limited to mitochondrial cytochrome oxidase subunit 1 (COI) gene sequences deposited to the NCBI database. Therefore, this new high-quality genome resource is valuable and will be useful for further studies, ranging from revealing their population structures, studying cross-kingdom interaction (e.g. insects/yeasts/plants), to understanding their evolution with other insects.

 This genome resource is excellent from the summary statistics, with high BUSCO number scores, high sequence continuity (scaffold N50), and majority of sequences contained on the 29 pseudochromosomes (plus sex chromosome and mitochondrion). All in all, this is another Darwin Tree of Life's valuable contribution.

Are sufficient details of methods and materials provided to allow replication by others?

Yes

Is the rationale for creating the dataset(s) clearly described?

Yes

Are the datasets clearly presented in a useable and accessible format?

Yes

Are the protocols appropriate and is the work technically sound?

Yes

Reviewer Expertise:

Genomics, evolution, invertebrates

I confirm that I have read this submission and believe that I have an appropriate level of expertise to confirm that it is of an acceptable scientific standard.

# Reviewer report: Reviewer response for version 1

**DOI:** 10.21956/wellcomeopenres.23522.r90424

**Published:** 2024-08-06

**Authors:** Annabel Whibley

**Affiliations:** 1 Bragato Research Institute, Lincoln, New Zealand; 2 The University of Auckland, Auckland, Auckland, New Zealand

In this Data Note, Boyes, Crowley, McCulloch, Boyes and colleagues present a reference genome assembly and gene annotation of the Elm Groundling Moth ( *Carpatolechia fugitivella).* The reference is high-quality, was constructed using appropriate tools and with comprehensive reporting of the sample collection, data generation and analysis and all associated metadata. Links to data accessions have been tested and are working.

**Minor comments:**

 Background:  “It is local in England and Wales” reads oddly to me. Perhaps “It is locally common/abundant”?

 Please specify longitude and latitude when giving co-ordinates.

 Please use consistent formatting for dates (Sample acquisition for one sample is “2020-08-01” and for  second is “2022-07-2”).

 I will continue to query whether this templated detail is correct: “in brief, the method employs a 1.8X ratio of AMPure PB beads to sample to eliminate shorter fragments and concentrate the DNA.”  This ratio of beads is not size-selective,  I believe this should be 0.6x, as in the protocols.io guidelines

Are sufficient details of methods and materials provided to allow replication by others?

Yes

Is the rationale for creating the dataset(s) clearly described?

Yes

Are the datasets clearly presented in a useable and accessible format?

Yes

Are the protocols appropriate and is the work technically sound?

Yes

Reviewer Expertise:

Genomics, Bioinformatics, Evolution

I confirm that I have read this submission and believe that I have an appropriate level of expertise to confirm that it is of an acceptable scientific standard.
